# Supplementary material for: Single-cell RNA sequencing in Hirschsprung’s disease tissues reveals lack of neuronal differentiation in the aganglionic colon segment
Source: bioRxiv. 2025 Jul 4:2025.07.01.662516. Preprint. [Version 1] doi: 10.1101/2025.07.01.662516 (PMC12236654; doi:10.1101/2025.07.01.662516)
Supplement: Supplement 1 [file media-1.pdf]

Single-cell RNA sequencing in Hirschsprung's disease tissues reveals lack of neuronal differentiation in the aganglionic colon segment

Szabolcs Tarapcsak[1], Xiaomeng Huang[1], Yi Qiao[1], Andrew Farrell[1], Lija Mammen[2], Amy Lovichik[3], Gayatri D. Khanderao[4], Teresa Musci[5], Philip J Moos[5], Matthew A Firpo[4], Michael Rollins[2], Gabor T Marth[1]

[1] *Eccles Institute of Human Genetics, University of Utah, Salt Lake City, Utah, USA*

[2] *Division of Pediatric Surgery, University of Utah Health, Salt Lake City, Utah, USA*

[3] *Department of Pathology, Primary Children's Hospital, Salt Lake City, Utah, USA*

[4] *Department of Surgery, University of Utah School of Medicine, Salt Lake City, Utah, USA*

[5] *Department of Pharmacology and Toxicology, College of Pharmacy, University of Utah, Salt Lake City, Utah, USA*

Corresponding author:

Gabor T. Marth,

Institute of Human Genetics

University of Utah School of Medicine

15 North 2030 East, Room 7410B Salt Lake City, UT 84112-5330

Phone: (801) 581-6158

Email: gmarth@genetics.utah.edu

The authors have declared that no conflict of interest exists.

SUPPLEMENTARY DATA

<u>Supplementary Table 1:</u> List of identified cell types in scRNAseq analysis and associated marker genes

**Cell type**	**Marker Genes:**
Enterocytes	*EPCAM+, KRT8+, KRT18+*
Goblet cells	*TFF3+, MUC2+, FCGPB+*
Endothelial cells	*PECAM1+, VWF+, CD93+*
Tuft cells	*TRPM5+, DCLK1+, GFI1B+*
Fibroblasts	*COL1A2+, COL3A1+, CFD+*
Smooth muscle cells	*ACTA2+, MYH11+, RGS5+*
B-cells	*IGHM+, CD79A+, CD83+, CXCR4+*
T-cells	*CD3D+, IL7R+, CD7+, CD2+*
Monocytes	*CD14+, LYZ+, IL1B+*
Mast cells	*KIT+, TPSAB1+, TBSB2+*
Neural progenitor cells	*NGFR+, UCHL1-, S100B-*
Mature neurons	*TUBB3+, UCHL1+, GAL+*
Mature glial cells	*S100B+, ERBB3+*

<u>Supplementary Table 2:</u> List of germline variants in HSCR-associated genes of HSCR patients

Accession numbers, status and condition is based on the ClinVar database. For variants that are not present in ClinVar, nucleotide change is provided (missense mutations).

	Gene	Type	Accession #	Status	Condition
Patient 1	DHCR7	Missense	VCV000093708	Benign	-
	RET	Synonymous	VCV000095995	Benign	-
		Synonymous	VCV000167590	Benign	-
	SEMA3D	Missense	-	-	c.2101A>C
	NRG1	Missense	-	-	c.866T>C
	NRG3	Synonymous	VCV000691408	Uncertain	Aganglionic megacolon
	EDNRB	Synonymous	VCV000226622	Benign	-
		Synonymous	VCV000226625	Benign	-
Patient 2	SEMA3D	Missense	-	-	c.2101A>C
	RET	Missense	VCV000024934	Conflicting pathogenicity	Hirschsprung's disease, Multiple endocrine neoplasia
	KIF1BP	Missense	-	-	c.196G>A
	DHCR7	Missense	VCV000093707	Benign	Smith-Lemli-Opitz syndrome, neurodevelopmental disorders
		Missense	VCV001177469	Benign	Smith-Lemli-Opitz syndrome, neurodevelopmental disorders
	TCF4	Missense	VCV000160083	Benign	Pitt-Hopkins Syndrome, Corneal dystrophy
Patient 3	DHCR7	Splice acceptor	VCV000093725	Pathogenic	Smith-Lemli-Opitz syndrome, neurodevelopmental disorders
		Missense	-	-	c.659T>C
		Missense	VCV001177469	Benign	Smith-Lemli-Opitz syndrome
	SEMA3D	Missense	-	-	c.2101A>C
	KIF1BP	Missense	-	-	c.196G>A
	NRG3	Missense	VCV000691406	Uncertain	Aganglionic megacolon
	GFRA1	Missense	VCV001328035	Benign	-

<u>Supplementary table 3:</u> List of germline variants in various signaling pathway receptor genes of HSCR patients

Accession numbers, status and condition is based on the ClinVar database.

	Gene	Type	Accession #	Status	Condition
Patient 1	NOTCH2	Missense	VCV000134972	Benign	Hajdu-Cheney Syndorme
		Missense	VCV000256152	Benign	Cerebral arteriopathy, Lateral meningocele syndrome
		Missense	VCV001181077	Benign	Cerebral arteriopathy, Lateral meningocele syndrome
		Missense	VCV000811011	Benign	Cerebral arteriopathy, Lateral meningocele syndrome
	NOTCH4	Missense	VCV001297197	Benign	-
		Missense	VCV001287249	Benign	-
		Missense	VCV001294880	Benign	-
	PTCH1	Missense	VCV000041663	Benign	Hereditary cancer-predisposing syndrome, Gorlin syndrome
	BMPR1A	Missense	VCV000041782	Benign	Juvenile polyposis syndrome
Patient 2	NOTCH4	Missense	VCV001220553	Benign	-
		Missense	VCV001297197	Benign	-
		Missense	VCV001287249	Benign	-
		Missense	VCV001294880	Benign	-
	NOTCH3	Missense	VCV000256152	Benign	Cerebral arteriopathy, Lateral meningocele syndrome
		Missense	VCV001181077	Benign	Cerebral arteriopathy, Lateral meningocele syndrome
		Missense	VCV000811011	Benign	Cerebral arteriopathy, Lateral meningocele syndrome
Patient 3	PTCH1	Missense	VCV000041663	Benign	Hereditary cancer-predisposing syndrome, Gorlin syndrome
	BMPR1A	Missense	VCV000041782	Benign	Juvenile polyposis syndrome
	NOTCH3	Missense	VCV000256152	Benign	Lateral meningocele syndrome, cerebral arteriopathy
		Missense	VCV001181077	Benign	Lateral meningocele syndrome, cerebral arteriopathy
		Missense	VCV000811011	Benign	Lateral meningocele syndrome, cerebral arteriopathy

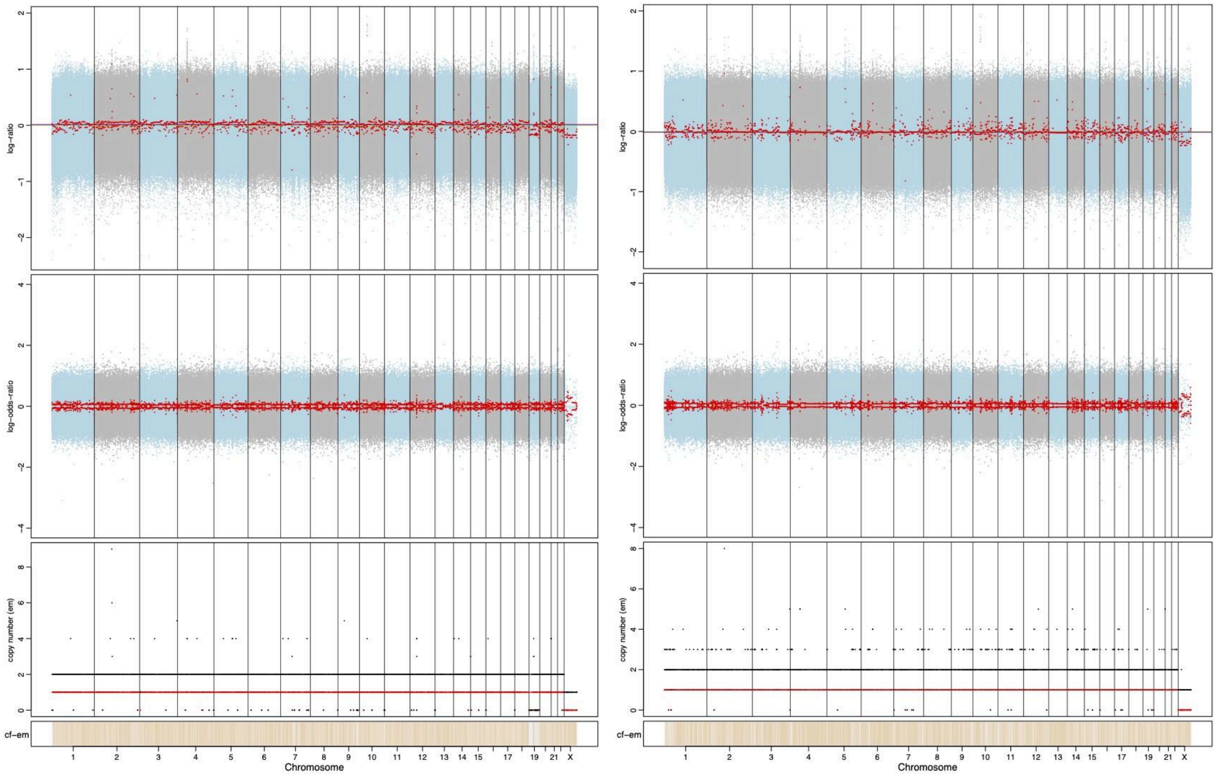

Supplementary Figure 1: FACETS output of Patient 1 healthy (left) and aganglionic (right) colon samples.

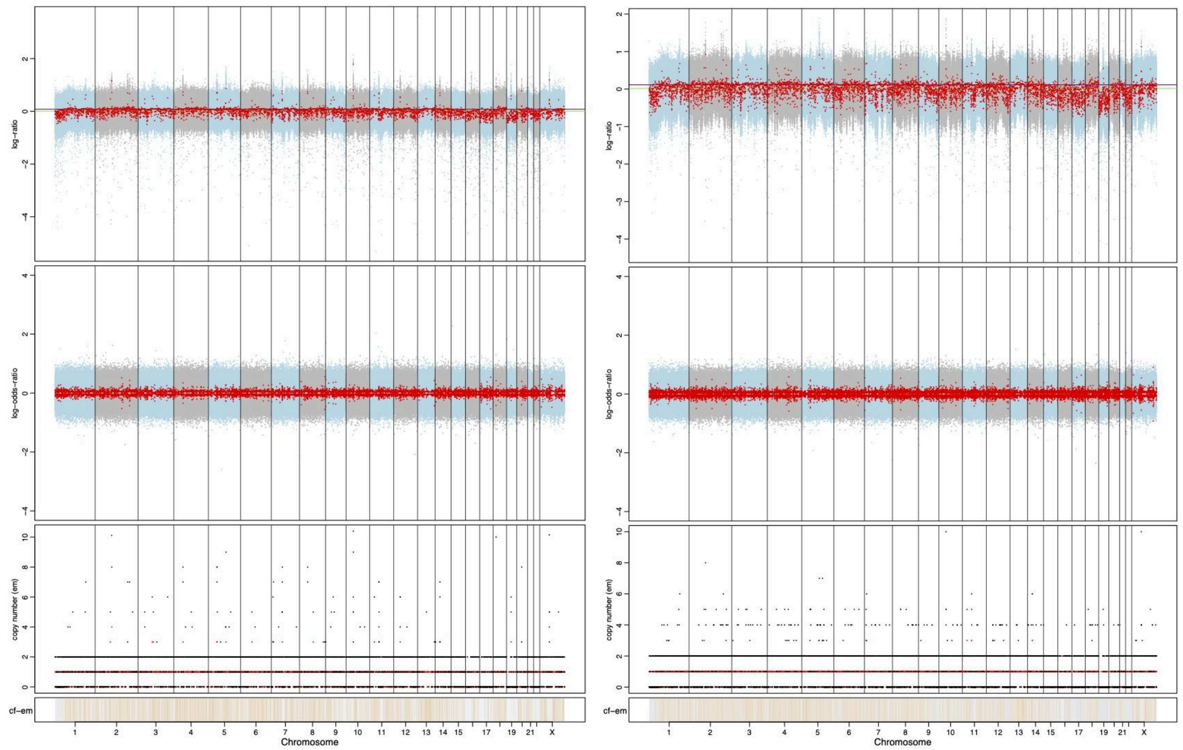

Supplementary Figure 2: FACETS output of Patient 2 healthy (left) and aganglionic (right) colon samples.

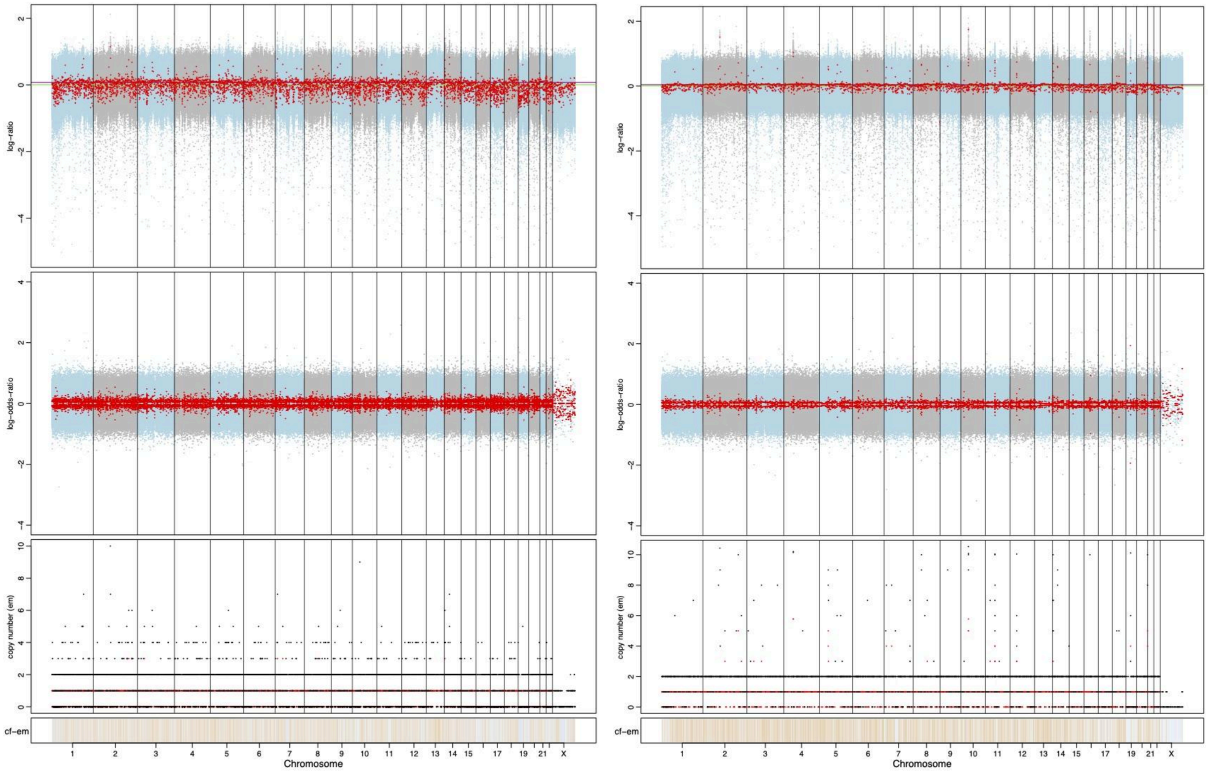

Supplementary Figure 3: FACETS output of Patient 3 healthy (left) and aganglionic (right) colon samples.

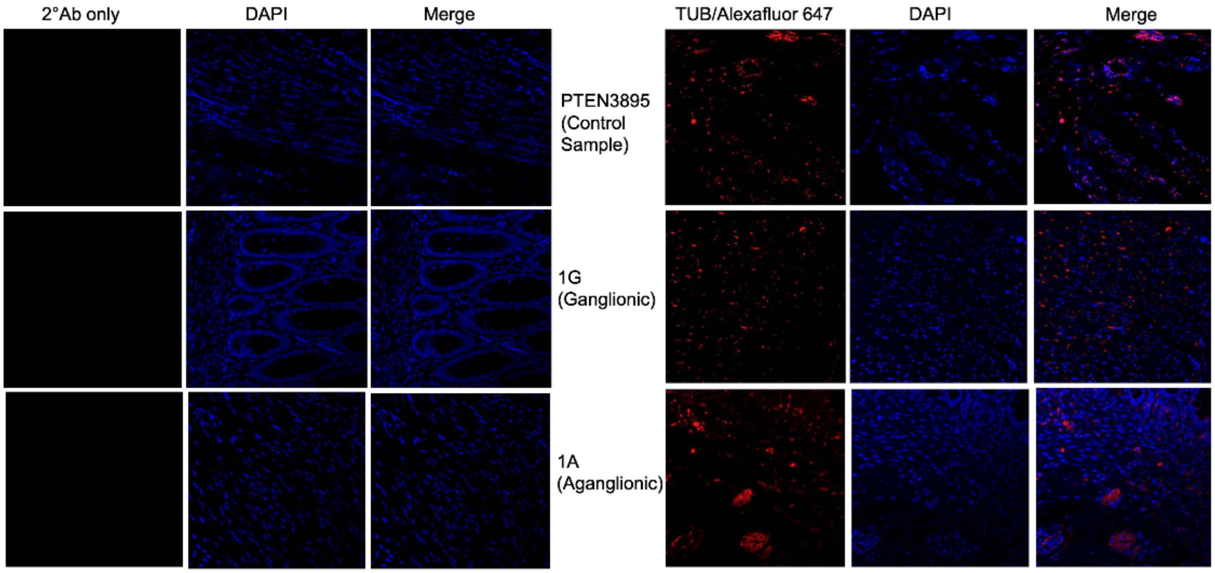

Supplementary Figure 4: Representative images from fluorescence microscopic analysis of HSCR healthy and aganglionic colon segments using DAPI and anti-p75 (*NGFR*) antibody staining. As positive control, PTEN3895 cells were used.
